# Supplementary material for: Task-oriented training in stroke rehabilitation: Qualitative study on perspectives and challenges among Pakistani physiotherapists
Source: PLoS One. 2025 Aug 20;20(8):e0330634. doi: 10.1371/journal.pone.0330634 (PMC12367181; doi:10.1371/journal.pone.0330634)
Supplement: S1 — (DOCX) [file pone.0330634.s001.docx]

**Semi-Structured Interview Questionnaire**

**Title: Task-Oriented Training in Stroke Rehabilitation – Perspectives of Physiotherapists**

**Section 1: Background Information**

1. Can you briefly describe your professional background and experience in stroke rehabilitation?
2. How many years have you been practicing as a physiotherapist?
3. What is your primary clinical setting (e.g., hospital, private clinic, rehabilitation center)?

**Section 2: Understanding of Task-Oriented Training (TOT)**

1. How would you define Task-Oriented Training (TOT) in stroke rehabilitation?
2. How frequently do you incorporate TOT in your rehabilitation sessions?
3. What factors influence your decision to use TOT with stroke patients?

**Section 3: Implementation of TOT in Motor Function Recovery**

1. What specific TOT techniques do you use for **upper limb rehabilitation**?
2. How do you adapt TOT for **lower limb rehabilitation**, particularly for gait and stepping exercises?
3. Have you encountered challenges in getting patients to engage in TOT for motor function recovery? If so, how do you address these challenges?

**Section 4: Cognitive Rehabilitation in TOT**

1. Do you integrate cognitive tasks into your TOT sessions? If yes, how?
2. What cognitive functions do you target (e.g., attention, memory, problem-solving)?
3. What challenges do you face in implementing cognitive components in TOT?

**Section 5: Balance Training and TOT**

1. How do you incorporate balance training within the TOT framework?
2. What balance-related challenges do stroke patients face, and how do you modify therapy accordingly?
3. What equipment or tools do you typically use for balance training?

**Section 6: Barriers and Challenges in Implementing TOT**

1. What are the **main challenges** you face in implementing TOT? (e.g., time constraints, patient compliance, lack of resources)
2. How do you address **resource limitations** when using TOT in your setting?
3. What strategies have you found effective in overcoming **patient-related barriers**, such as fear of movement or low motivation?

**Section 7: Cultural and Contextual Influences**

1. How do cultural beliefs and patient expectations influence the use of TOT in your practice?
2. Do socioeconomic factors (e.g., affordability, access to rehabilitation centers) affect patient participation in TOT?
3. What adaptations do you make to ensure TOT is accessible and relevant for diverse patient populations?

**Section 8: Future Recommendations**

1. What improvements would you suggest for better integrating TOT in stroke rehabilitation in Pakistan?
2. What additional training or resources would help physiotherapists implement TOT more effectively?
3. Is there anything else you would like to add regarding your experience with TOT?
