# Supplementary material for: Task-oriented training in stroke rehabilitation: Qualitative study on perspectives and challenges among Pakistani physiotherapists
Source: PLoS One. 2025 Aug 20;20(8):e0330634. doi: 10.1371/journal.pone.0330634 (PMC12367181; doi:10.1371/journal.pone.0330634)
Supplement: S2 — (DOCX) [file pone.0330634.s002.docx]

**EXCERPTS OF THE TRANSCRIPTS**

Demographic details

| **Demographics** | | **Frequencies n (%)** |
| --- | --- | --- |
| **Gender** | Male | n=15 (68.2%) |
|  | Female | n=7 (31.8%) |
| **Cities** | Peshawar | n=2 (9.1%) |
|  | Lahore | n=11 (50.0%) |
|  | Islamabad | n=2 (9.1%) |
|  | Rawalpindi | n=1 (4.5%) |
|  | Jhelum | n=1 (4.5%) |
|  | Faisalabad | n=5 (22.7%) |
| **Age (Years)** | 35.09 + 2.81 (Mean + Std. Deviation) | |
| **Years of Experience** | 5.45 + 0.59 (Mean + Std. Deviation) | |

**Percentages of participant’s responses to themes and sub themes:**

| **Theme** | **Sub-Theme** | **n** | **%** |
| --- | --- | --- | --- |
| **1. TOT Practices** | Patient-Centered Approach | 18 | 82% |
|  | Meaningful Activity Integration | 18 | 82% |
| **2. Motor Function Improvement** | Upper Limb Rehabilitation | 18 | 82% |
|  | Lower Limb Rehabilitation | 18 | 82% |
| **3. Cognitive Rehabilitation** | Emphasis on Cognitive Rehabilitation | 8 | 36% |
|  | • Attention Training | 7 | 32% |
|  | • Quantitative Reasoning | 8 | 36% |
|  | • Memory Retention | 8 | 36% |
| **4. Balance Training** | Static Balance | 18 | 82% |
|  | Dynamic Balance | 18 | 82% |
| **5. Challenges & Barriers in Implementing TOT** | Resource Constraints | 15 | 68% |
|  | Time Constraints | 12 | 55% |
|  | Knowledge Gaps & Standardization | 10 | 45% |
|  | Patient-Related Barriers | 10 | 45% |
| **6. Cultural & Contextual Factors** | Cultural Norms & Values | 12 | 55% |
|  | Healthcare System Factors | 11 | 50% |
|  | Socioeconomic Factors | 10 | 45% |
|  | Language & Communication | 7 | 32% |

**Code book**

| Code_Name | Definition |
| --- | --- |
| Patient_Centered_Approach | Therapy tailored to patient’s individual goals and contexts. |
| Meaningful_Activity_Integration | Use of real-life activities to make therapy meaningful. |
| Upper_Limb_Rehabilitation | Gross motor tasks like reaching, lifting objects. |
| Lower_Limb_Rehabilitation | Lower limb tasks like stepping, gait training. |
| Attention_Training | Dual-task exercises to improve attention. |
| Quantitative_Reasoning | Tasks involving numerical problem-solving. |
| Memory_Retention | Exercises aimed at improving memory recall. |
| Static_Balance | Exercises focused on static postural stability. |
| Dynamic_Balance | Exercises to improve balance during movement. |
| Resource_Constraints | Lack of equipment or funding for TOT. |
| Time_Constraints | Insufficient time per patient session. |
| Knowledge_Gaps_and_Standardization | Gaps in formal training and lack of standards. |
| Patient_Related_Barriers | Barriers related to patient motivation and cognition. |
| Cultural_Norms_and_Values | Cultural beliefs and values influencing rehab. |
| Healthcare_System_Factors | Healthcare infrastructure and policy constraints. |
| Socioeconomic_Factors | Financial and socioeconomic access issues. |
| Language_and_Communication | Language barriers and need for visual aids. |

**Interview transcripts excerpts**

**Transcript 1 (Participant P1, Urban Clinic)**

**Interviewer (I):** Thank you for speaking with me today. To begin, how do you define task-oriented training in your clinical practice?
**P1:** TOT for me means “using real-life tasks—like brushing teeth or carrying a plate—instead of isolated exercises. It makes therapy relevant and motivates patients.”

**I:** Which motor activities do you most often employ for upper-limb rehabilitation?
**P1:** “I start with gross movements—reaching and lifting light objects. Once they regain strength, we progress to fine motor tasks, such as picking up coins or buttoning a shirt.”

**I:** Have you integrated any cognitive tasks into these sessions?
**P1:** “Occasionally. About a third of my patients do a dual-task challenge, like naming days of the week backward while walking. But I find many lack patience for it.”

**I:** What barriers do you face when implementing TOT?
**P1:** “Resource constraints are big—no balance boards or robotics here. Also, with 10–12 patients a day, time is very limited for thorough TOT.”

**I:** How do cultural or socioeconomic factors influence therapy?
**P1:** “Many families prefer herbal remedies first. And if patients can’t take a day off work, they skip sessions. I often teach home-based exercises they can fit into their routine.”

**Transcript 2 (Participant P8, Semi-Urban Clinic)**

**I:** Can you describe how you make your rehabilitation sessions meaningful for patients?
**P8:** “I ask patients what they struggle with at home—like carrying groceries—then recreate that in the clinic. It helps them see the purpose of each exercise.”

**I:** Tell me about your approach to balance training.
**P8:** “We do single-leg stands and tandem walking in the hallway. If we lack equipment, I set up cushions or foam pads for uneven-surface practice.”

**I:** What kind of training did you receive for TOT?
**P8:** “Honestly, very little formal training. Most of what I know comes from workshops and self-study. A standardized protocol would help everyone.”

**I:** Have language or communication issues ever posed a challenge?
**P8:** “Yes—some patients speak only regional dialects. I rely on demonstrations, pictures, and simple gestures to ensure they understand each task.”

**Transcript 3 (Participant P14, Rural Outreach)**

**I:** In your view, what is the biggest obstacle to using TOT with lower-limb patients?
**P14:** “Fear of falling is huge. Many patients refuse to stand or walk at first. I start with support belts and bedside stepping exercises to build confidence.”

**I:** Do you involve caregivers in the training process?
**P14:** “Always. I show family members how to guide exercises at home—this increases adherence when patients lack tech or travel support.”

**I:** How do you address cognitive training needs in stroke survivors?
**P14:** “I break tasks into small steps and use visual cues—like colored cards for memory drills. Still, only about 4 of my 22 patients consistently engage in cognitive tasks.”
