# Supplementary material for: Task-oriented training in stroke rehabilitation: Qualitative study on perspectives and challenges among Pakistani physiotherapists
Source: PLoS One. 2025 Aug 20;20(8):e0330634. doi: 10.1371/journal.pone.0330634 (PMC12367181; doi:10.1371/journal.pone.0330634)
Supplement: S3 — (DOCX) [file pone.0330634.s003.docx]

S1 Table: Coding Tree for Task-Oriented Training in Stroke Rehabilitation

| **Main Theme** | **Sub-Themes** | **Codes** | **Negative Cases / Barriers** | **Adaptation Strategies / Facilitators** |
| --- | --- | --- | --- | --- |
| **1. Task-Oriented Training (TOT) Practices** | Patient-Centered Approach | Functional relevance, goal-oriented therapy, holistic rehabilitation | Lack of standardized TOT protocols | Therapists adapt based on training & available resources |
|  | Meaningful Activity Integration | Reaching for objects, standing exercises, walking on different surfaces | Inconsistent practice among therapists | Individualized task selection |
| **2. Motor Function Improvement** | Upper Limb Rehabilitation | Gross motor tasks (reaching, lifting), fine motor control (grasping, buttoning) | Limited equipment for fine motor training | Use of everyday objects for therapy |
|  | Lower Limb Rehabilitation | Stepping exercises, gait training, stair climbing | Patient fear of falling limits adherence | Gradual progression & caregiver support |
| **3. Cognitive Rehabilitation** | Attention Training | Dual-task exercises (e.g., mental calculations while walking) | Low patient motivation, frustration | Breaking tasks into smaller steps, visual prompts |
|  | Quantitative Reasoning | Handling money, problem-solving exercises | Some patients struggle with cognitive tasks | Functional, real-life simulations |
|  | Memory Retention | Object recall games, naming sequences | Difficulty in engaging patients with cognitive impairments | Caregiver involvement in therapy |
| **4. Balance Training** | Static Balance | Single-leg stands, tandem stance training | Lack of specialized balance equipment | Improvised balance training with household items |
|  | Dynamic Balance | Obstacle navigation, walking on uneven terrain | Limited access to VR-based balance rehabilitation | Substituting with step-over exercises |
| **5. Challenges and Barriers in Implementing TOT** | Resource Constraints | Lack of TOT-specific equipment, funding shortages | Patients in rural areas lack access to rehab | Home-based modifications, alternative tools |
|  | Time Constraints | TOT is time-intensive for therapists with high patient loads | Limited session duration affects implementation | Prioritization of functional tasks |
|  | Knowledge Gaps & Standardization | Variability in TOT application due to lack of formal training | Inconsistent implementation across clinics | Suggested TOT workshops for physiotherapists |
|  | Patient-Related Barriers | Fear of movement, low motivation, cognitive impairments | Patients stop therapy due to slow progress | Goal-setting, family support, real-life task demonstration |
| **6. Cultural and Contextual Factors** | Cultural Norms and Values | Traditional beliefs influence patient adherence | Preference for rest & herbal remedies over physiotherapy | Family integration improves adherence |
|  | Healthcare System Factors | Urban-centric rehab services, lack of insurance coverage | Limited access to facilities in rural areas | Development of low-cost home-based TOT programs |
|  | Socioeconomic Factors | Financial constraints, limited rehab funding | Patients skip therapy due to work commitments | Cost-effective home exercises with no equipment |
|  | Language and Communication | Language barriers affect understanding of therapy | Rural patients struggle with Urdu instructions | Use of non-verbal communication & visual aids |

S2 Table: Thematic Saturation Tracking

| **Interview Number** | **New Themes Identified** | **New Sub-Themes Identified** | **Recurring Themes/Sub-Themes** | **Saturation Status** |
| --- | --- | --- | --- | --- |
| **Interview 1** | Task-Oriented Training Practices | Patient-centered approach, meaningful activity integration | - | Not reached |
| **Interview 2** | Motor Function Improvement | Upper & lower limb rehabilitation | TOT Practices | Not reached |
| **Interview 3** | Cognitive Rehabilitation | Attention training, memory retention | TOT, Motor Function | Not reached |
| **Interview 4** | Balance Training | Static vs. dynamic balance | TOT, Motor Function, Cognitive | Not reached |
| **Interview 5** | Challenges in Implementing TOT | Resource constraints, time limitations | Previous themes reinforced | Not reached |
| **Interview 6** | Cultural and Contextual Factors | Traditional beliefs, healthcare system barriers | All previous themes reinforced | Emerging saturation |
| **Interview 7** | - | Language barriers, socioeconomic factors | All previous themes reinforced | Emerging saturation |
| **Interview 8** | - | Cognitive engagement barriers | All previous themes reinforced | Near saturation |
| **Interview 9** | - | - | No new themes/sub-themes emerged | Near saturation |
| **Interview 10** | - | - | No new themes/sub-themes emerged | Near saturation |
| **Interview 11** | - | - | No new themes/sub-themes emerged | Near saturation |
| **Interview 12** | - | - | No new themes/sub-themes emerged | Near saturation |
| **Interview 13** | - | - | No new themes/sub-themes emerged | Near saturation |
| **Interview 14** | - | - | No new themes/sub-themes emerged | Near saturation |
| **Interview 15** | - | - | No new themes/sub-themes emerged | Near saturation |
| **Interview 16** | - | - | No new themes/sub-themes emerged | Near saturation |
| **Interview 17** | - | - | No new themes/sub-themes emerged | Near saturation |
| **Interview 18** | - | - | No new themes/sub-themes emerged | Near saturation |
| **Interview 19** | - | - | No new themes/sub-themes emerged | Last unique insights captured |
| **Interview 20** | - | - | No new themes/sub-themes emerged | Thematic Redundancy Reached |
| **Interview 21** | - | - | No new themes/sub-themes emerged | Confirming Saturation |
| **Interview 22** | - | - | No new themes/sub-themes emerged | Confirming Saturation |

S3 table: percentages and frequencies of participants responses to themes and subthemes

| **Theme** | **Sub-Theme** | **n** | **%** |
| --- | --- | --- | --- |
| **1. TOT Practices** | Patient-Centered Approach | 18 | 82% |
|  | Meaningful Activity Integration | 18 | 82% |
| **2. Motor Function Improvement** | Upper Limb Rehabilitation | 18 | 82% |
|  | Lower Limb Rehabilitation | 18 | 82% |
| **3. Cognitive Rehabilitation** | Emphasis on Cognitive Rehabilitation | 8 | 36% |
|  | • Attention Training | 7 | 32% |
|  | • Quantitative Reasoning | 8 | 36% |
|  | • Memory Retention | 8 | 36% |
| **4. Balance Training** | Static Balance | 18 | 82% |
|  | Dynamic Balance | 18 | 82% |
| **5. Challenges & Barriers in Implementing TOT** | Resource Constraints | 15 | 68% |
|  | Time Constraints | 12 | 55% |
|  | Knowledge Gaps & Standardization | 10 | 45% |
|  | Patient-Related Barriers | 10 | 45% |
| **6. Cultural & Contextual Factors** | Cultural Norms & Values | 12 | 55% |
|  | Healthcare System Factors | 11 | 50% |
|  | Socioeconomic Factors | 10 | 45% |
|  | Language & Communication | 7 | 32% |
