# Supplementary material for: Task-oriented training in stroke rehabilitation: Qualitative study on perspectives and challenges among Pakistani physiotherapists
Source: PLoS One. 2025 Aug 20;20(8):e0330634. doi: 10.1371/journal.pone.0330634 (PMC12367181; doi:10.1371/journal.pone.0330634)
Supplement: Attachment — Submitted filename: Response to Reviewers.docx [file pone.0330634.s005.docx]

**Journal requirements:**

1.  Please ensure that your manuscript meets PLOS ONE's style requirements, including those for file naming.

**Answer:** Thank you for pointing this out. We have reformatted the entire manuscript using the PLOS ONE style templates for both the main body and the title/author page. All files have been renamed according to the journal’s naming convention (e.g., “Manuscript.docx,” “Response_to_Reviewers.docx,” “Revised_Manuscript_Track_Changes.docx,” etc.). We trust that these changes now fully comply with PLOS ONE’s formatting requirements.

2. We note that you have indicated that there are restrictions to data sharing for this study. PLOS only allows data to be available upon request if there are legal or ethical restrictions on sharing data publicly. If there are no restrictions, please upload the minimal anonymized data set necessary to replicate your study findings to a stable, public repository and provide us with the relevant URLs, DOIs, or accession numbers. For a list of recommended repositories, please see

<https://journals.plos.org/plosone/s/recommended-repositories> . You also have the option of uploading the data as Supporting Information files, but we would **recommend depositing data directly to a data repository if possible**

**Answer:** We have prepared a Supporting Information file, , which provides anonymized, thematically organized excerpts illustrating each major theme and sub-theme. This file has been uploaded with our revision

**Reviewer 1

Abstract**

1. Abbreviate Task-Oriented Training as TOT upon its first mention and use the abbreviation consistently throughout the manuscript.

**Answer:** Thank you for your valuable feedback. We have abbreviated Task-Oriented Training as TOT upon its first mention in the manuscript and have used the abbreviation TOT consistently throughout the text to enhance clarity and maintain consistency.

2. The results section of the abstract may include numerical values derived from the data analysis (e.g., percentage of therapists using TOT, common barriers identified, response rates) as applicable

**Answer:** Thank you for this helpful suggestion. We have revised the Results section of the Abstract to include both raw numbers and percentages, specifying the number of therapists who incorporated TOT, emphasized cognitive rehabilitation, and identified major barriers. We have also included table for all themes and subthemes percentages reported by participants

**Introduction**

3. Replace vague expressions like “with the rising incidence of stroke in LMICs” with quantitative epidemiological data, citing specific incidence or prevalence rates.

**Answer:** Thank you for this recommendation. We have replaced the vague expression with up-to-date, quantitative data on Pakistani‐specific stroke burden.

4. The statement “influences survivors' ability to resume work” should specify the age group most affected, supported by relevant literature.

**Answer:** Thank you for this suggestion. We have revised the sentence to specify the working-age group most affected and added a supporting citation in next sentence.

5. Clarify or provide citation evidence for the following claim:

“However, while TOT has been extensively considered in Western contexts of rehabilitation, its application in LMICs, particularly in Pakistan, remains underexplored.”

Include region-specific studies or a literature gap analysis to support this assertion.

**Answer:** We thank the reviewer for highlighting the need to substantiate this claim. We have revised the sentence to cite both robust evidence from high-income countries and the scarcity of Pakistan-specific research

**References**

6. Reference no. 10 is not accessible—please provide a valid source or replace with an alternative that is publicly available.

**Answer:** We thank the reviewer for noting this issue. We have replaced the original citation with a fully accessible, open-access article from the Insights Journal of Health and Rehabilitation. The revised reference is as follows:

**10. **Rasool A, Tahir Z, Mukhtar I, Fatima M, Riaz Z, Hussain F. Comparison of competency levels among physical therapists practicing in public and private health care centers of Faisalabad, Pakistan. Insights Journal of Health and Rehabilitation. 2024;2(2):163–169. Available from: <https://insightsjhr.com/index.php/home/article/view/118>

7. Ensure all citations used to support methodology or clinical practice (especially those describing rehabilitation procedures) are appropriately cited.

**Answer:** We have carefully reviewed the manuscript to confirm that every methodological decision and clinical practice statement is now supported by an appropriate, peer-reviewed citation.

**Methodology:**

8. Sample Size: Justify why only 22 therapists were selected for purposive sampling. Explain whether saturation was achieved.

**Answer:** Thank you for flagging this. We have indeed clarified our sampling rationale and saturation process in the Materials and Methods section under “Sample Size and Participant Characteristics. Specifically, we note that purposive sampling targeted physiotherapists with ≥5 years of stroke‐rehabilitation experience across major Pakistani cities, and that data collection continued until saturation was reached by interview 20, with two additional interviews confirming no new themes. For ease of reference, we have now added a cross‐reference to the Saturation Table in the supporting information file.

**Questionnaire Validation: Clearly state:**

- How many therapists were involved in validating the semi-structured interview tool?
- Their qualifications and professional background.
- Whether these therapists had prior knowledge or experience with TOT (as part of inclusion criteria).
  - Language of Questionnaire: Specify the language in which the questionnaire was developed and administered.
  - Content Validation: Confirm if the tool explicitly assesses whether the participants are acquainted with the TOT approach.

**Answer:** We thank the reviewer for this request for clarification. We have expanded the Materials and Methods section to include the following details about questionnaire validation:

**Questionnaire Validation**

The semi-structured interview guide was validated by three independent expert physiotherapists—each holding a Master’s degree in Neuro-Muscular Physical Therapy and with ≥7 years of clinical experience in stroke rehabilitation, including prior use of task-oriented training (TOT). These experts reviewed the tool for clarity, relevance, and cultural appropriateness, and confirmed that all items effectively assess clinicians’ familiarity with and application of TOT.

**Language of Administration**

The questionnaire was drafted and administered in English, the medium of instruction and clinical communication for all participants. Where necessary, interviewers provided Urdu explanations for technical terms to ensure comprehension.

**Content Validation**

As part of the guide, the first section included an item explicitly asking participants to describe their prior training and experience with TOT, thereby confirming their acquaintance with the approach before proceeding to detailed questions.

**Data Analysis**

• Report response rates across different sections/domains of the semi-structured questionnaire.

• Include any correlation or association found between participant characteristics (e.g., experience level, clinical setting) and the use or understanding of TOT.

• Ensure the similarity index of the manuscript is <10% and revise any plagiarized or overly similar content from prior publications.

**Answer:** We appreciate the reviewer’s guidance. We have made the following changes:

**Questionnaire Response Rates:**

In the result section, we now report that all 22 participants (100%) completed each section of the semi-structured guide (demographics, TOT practices, motor function, cognitive rehabilitation, balance training, barriers, and cultural contexts). The frequencies of specific thematic responses are detailed in S3 Table.

**Participant Characteristics and TOT Use:**

We did explore the analysis of associations between years of clinical experience (<6 vs. ≥6 years) and practice setting (urban vs. semi-urban) with TOT uptake. No clear patterns emerged: physiotherapists across all experience levels and settings reported similar integration of TOT, suggesting widespread adoption irrespective of background. And because of word count limitations we did not add it in manuscript.

**Similarity Index:**

**Answer**: We screened the revised manuscript using Turnitin and obtained a similarity index of 2%. All overlapping text has been rephrased or properly cited to ensure originality.

**Results**

- Present all findings using numerical values, e.g.,
- “68% of therapists use TOT in upper limb rehabilitation”
- “Only 27% of therapists incorporate cognitive tasks into sessions”
  - When reporting themes from qualitative analysis, quantify the responses where possible (e.g., “12 out of 22 participants identified lack of training as a barrier”).

**Answer:** Thank you. We have revised the Results section to include both raw numbers and percentages for every theme and sub-theme

**Discussion**

• Begin the discussion section with a summary of key findings.

• Compare your findings with existing studies or systematic reviews, particularly from LMICs or similar rehabilitation settings.

• Offer explanations for any discrepancies between your findings and prior research.

• Discuss the strength of the evidence supporting TOT’s effectiveness, referencing high-quality studies (e.g., RCTs, meta-analyses) that support its use in stroke rehabilitation.

**Answer:** Thank you for these valuable suggestions. We have thoroughly revised the Discussion

**Reviewer #2: Strengths:**

Suggestions for Improvement:

1. Consider slightly condensing some repetitive statements in the introduction and discussion for improved readability.

**Answer:** Thank you for this suggestion. We have reviewed both the Introduction and Discussion for repetitive phrasing and have condensed overlapping statements. In the Introduction, we streamlined background descriptions by merging similar sentences on stroke burden and rehabilitation needs. In the Discussion, redundant comparisons and explanatory passages were consolidated into more focused paragraphs, improving overall readability and narrative flow.

2. Some grammatical and syntax refinements (minor) are recommended; for instance, changing “has extensively reported” to “have extensively reported” (line 91) for subject-verb agreement.

**Answer:** Thank you for highlighting these grammatical and syntax issues. We have carefully proofread the manuscript and made the corrections

3. A brief table summarizing key themes and subthemes with representative quotes would enhance the clarity of findings.

**Answer:** Thank you for this excellent suggestion. We have already added Table for themes, subthemes, with coding tree.
