# Supplementary material for: Task-oriented training in stroke rehabilitation: Qualitative study on perspectives and challenges among Pakistani physiotherapists
Source: PLoS One. 2025 Aug 20;20(8):e0330634. doi: 10.1371/journal.pone.0330634 (PMC12367181; doi:10.1371/journal.pone.0330634)
Supplement: Attachment — Submitted filename: Response_to_Reviewers_auresp_2.docx [file pone.0330634.s006.docx]

**Response to Reviewers**

- **Journal Requirements:**

  **Please review your reference list to ensure that it is complete and correct. If you have cited papers that have been retracted, please include the rationale for doing so in the manuscript text, or remove these references and replace them with relevant current references. Any changes to the reference list should be mentioned in the rebuttal letter that accompanies your revised manuscript. If you need to cite a retracted article, indicate the article’s retracted status in the References list and also include a citation and full reference for the retraction notice.**

**Response:** We have thoroughly reviewed all references in our manuscript. None of the cited sources are currently listed as retracted, based on searches in PubMed and the Retraction Watch Database as of June 2025. Additionally, missing reference details have been completed, and formatting was corrected to conform to journal style guidelines.

- **Reviewer #1: The description of the sampling method is generally clear; however, a few points require clarification and elaboration. First, while purposive sampling is for targeting experienced professionals, the rationale behind selecting only physiotherapists with a Master’s degree in Neuro-Muscular Physical Therapy should be explained more explicitly. Clarifying whether this criterion was intended to ensure a homogeneous expert group or to reflect a standard qualification in the region would strengthen the methodological justification. Is it intended in inclusion criteria??**

**Response:** Thank you for this important observation. We have now clarified in the Materials and Methods section (under “Sample Size and Participants Characteristics”) that the selection criterion was intended to ensure a homogeneous expert group with specialized training in neurorehabilitation. This degree represents the standard qualification for clinical specialization in stroke rehabilitation in the Pakistani context.

- **Additionally, while the recruitment sources are diverse, it would be helpful to discuss any potential selection biases that may arise from relying on professional associations and academic networks. Please comment on whether this urban and semi-urban focus was intentional and how it may affect the generalizability of findings.**

**Response:** We thank the reviewer for this insightful observation. We acknowledge the potential for selection bias arising from our recruitment strategy, which relied on professional associations and academic networks. This approach may have limited participation from therapists working in rural or informal settings. However, the focus on urban and semi-urban participants was intentional, as these areas represent the principal hubs for structured neurorehabilitation services in Pakistan. Consequently, the findings are most representative of physiotherapists operating in formal clinical environments where task-oriented training is more feasibly implemented.

- **Reviewer #1 suggested including brief information on how many participants were expected from each recruitment source to clarify sample composition.**

**Response:** We appreciate this suggestion. While we did not set predefined quotas for recruitment sources, participants were drawn proportionally based on availability and responsiveness from each channel. Approximately 40% of the participants were recruited through professional physiotherapy associations, 35% through academic networks, and 25% via direct referrals from rehabilitation centers. This distribution reflects the practical accessibility of experienced neurorehabilitation professionals within the urban and semi-urban clinical ecosystem.

- **Reviewer #2 commended the manuscript for its content, discussion, and methodological rigor.**

**Response:** We sincerely thank Reviewer #2 for the encouraging feedback and appreciation of the revisions. We are pleased that the methodological clarity and depth of discussion meet the expectations and contribute meaningfully to the literature on task-oriented training in stroke rehabilitation.
